# AD16 Modulates Microglial Activation and Polarization to Mitigate Neuroinflammation in Ischemic Stroke Models Through α7nAChR‐ERK‐STAT3 Signaling

**DOI:** 10.1111/cns.70519

**Published:** 2025-07-25

**Authors:** Guo‐Jian Zhao, Li‐Mei Zhang, Si‐Rou Wang, Mei Yang, Jia‐Hao Jiang, Bo‐Xiang Yuan, Cheng Huang, Zhi‐Hua Huang, Xiao‐Lu Tang, Tao Chen

**Affiliations:** ^1^ Jiangxi Province Key Laboratory of Pharmacology of Traditional Chinese Medicine, Key Laboratory of Prevention and Treatment of Cardiovascular and Cerebrovascular Diseases of Ministry of Education, Ganzhou Key Laboratory of Neuroinflammation Research Gannan Medical University Ganzhou China; ^2^ College of Physical Education and Health Science, Yibin University Yibin China; ^3^ School of Basic Medicine Sciences Gannan Medical University Ganzhou China; ^4^ The First Clinical College of Gannan Medical University Ganzhou China

**Keywords:** α7nAChR‐ERK‐STAT3, AD16, ischemic stroke, microglia polarization, neuroinflammation

## Abstract

**Background:**

Neuroinflammation constitutes a critical pathological event subsequent to ischemic stroke. AD16, a novel anti‐neuroinflammatory compound, has demonstrated efficacy in alleviating neuroinflammation in neonatal rats induced by ischemia–hypoxia. This study aims to elucidate the therapeutic utility and underlying mechanisms of AD16 in an adult ischemic stroke rat model.

**Methods:**

A rat transient middle cerebral artery occlusion (tMCAO) model was employed. Neurological function was evaluated using the Longa and Garcia JH scores, motor function was assessed through rotary rod and CatWalk gait analysis, and brain injury was examined via TTC and Nissl staining. Molecular docking techniques simulate the binding of a target compound to a potential target. Western blot, immunofluorescence, and enzyme‐linked immunosorbent assay (ELISA) were used to detect microglia phenotype, pro‐inflammatory factors, and activation of signaling molecules.

**Results:**

AD16 treatment improved neural function in tMCAO rats, reduced cerebral infarction volume and brain water content, preserved blood–brain barrier integrity, and inhibited pro‐inflammatory cytokines. Molecular docking showed AD16 has high affinity for α7nAChR, TLR4, ERK, and STAT3. AD16 increased α7nAChR, CD206, and p‐ERK protein levels, while decreasing CD40, CD68, TLR4, and p‐STAT3. These effects were reversed by α‐BTX (α7nAChR inhibitor) and U0126 (ERK inhibitor).

**Conclusion:**

AD16 may inhibit microglia activation and polarization via the α7nAChR‐ERK‐STAT3 pathway, thus reducing neuroinflammation from cerebral ischemia and protecting the brain. This study suggests AD16 as a potential treatment for ischemic stroke.

## Graphical abstract

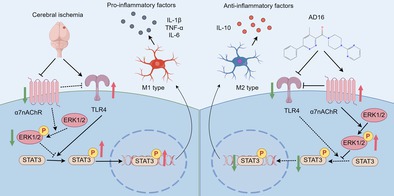

**Abbreviations**
AD: Alzheimer's disease
ANOVA: analysis of variance
BBB: blood–brain barrier
CAP: cholinergic anti‐inflammatory pathway
CCA: common carotid artery
CI/RI: cerebral ischemia/reperfusion injury
CNS: central nervous system
DMSO: dimethyl sulfoxide
ECA: external carotid artery
ELISA: enzyme‐linked immunosorbent assay
ERK: extracellular signal regulated kinase
ICA: internal carotid artery
IL‐10: interleukin‐10
IL‐1β: interleukin‐1β
IL‐6: interleukin‐6
LPS: lipopolysaccharide
NF‐κB: nuclear factor‐kappaB
NRF2: nuclear factor E2 related factor 2
STAT3: signal transducer and activator of transcription 3
tMCAO: transient middle cerebral artery occlusion
TNF‐α: tumor necrosis factor α
TTC: 2,3,5‐triphenyltetrazolium chloride
α7nAChR: α7 nicotinic acetylcholine receptor
α‐BTX: alpha‐bungarotoxin

## 1 Introduction

Stroke, mainly ischemic (85%), is a major health threaten [1]. The FDA‐approved treatment, tissue plasminogen activator, must be given within 4.5 h [2]. Blood flow restoration can cause cerebral ischemia–reperfusion injury (CI/RI), leading to irreversible brain damage from oxidative stress, amino acid toxicity, inflammation, and apoptosis [3]. Neuroinflammation is crucial in this process, making inflammation inhibition key to CI/RI treatment.

Microglia, resident immune cells of the central nervous system (CNS) that maintain brain homeostasis and trigger immune responses and inflammation [4]. Post‐ischemic stroke, they rapidly activate and differentiate into pro‐inflammatory (M1) and anti‐inflammatory (M2) types [5]. M1 microglia release pro‐inflammatory factors like tumor necrosis factor α (TNF‐α), interleukin‐6 (IL‐6) and interleukin‐1β (IL‐1β), which worsen neuronal damage, while M2 microglia secrete interleukin‐10 (IL‐10) and other anti‐inflammatory cytokines to aid brain repair [6]. Thus, reducing M1 polarization or enhancing M2 polarization to increase the M2/M1 ratio can help mitigate neuroinflammation caused by cerebral ischemia.

The cholinergic anti‐inflammatory pathway (CAP) serves as a physiological mechanism within the central nervous system that modulates immune responses and inflammation [7]. The α7 nicotinic acetylcholine receptor (α7nAChR) is integral to the functioning of CAP, and its activation has been shown to markedly attenuate inflammatory responses both in vivo and in vitro, positioning it as a promising therapeutic target for inflammatory diseases [8]. Extensive research indicates that α7nAChR activation can inhibit M1‐type microglial polarization while promoting M2‐type polarization, thereby mitigating neuroinflammation subsequent to ischemic stroke [9, 10].

Signal Transducer and Activator of Transcription 3 (STAT3) is a pivotal transcription factor involved in the regulation of immune responses and inflammatory processes [11]. Research shows that melatonin modulates the polarization of microglia towards M2 type via the STAT3 signaling pathway in ischemic stroke treatment [12]. Meanwhile, studies indicate that the Extracellular signal‐regulated kinases (ERKs) signaling pathway is key in regulating microglia M1/M2 polarization, reducing neuroinflammation and cerebral ischemia/reperfusion injury (CI/RI) in rat models [13, 14]. According to a recent report, the ERK/STAT3 signaling pathway helps modulate microglial polarization, improving lipopolysaccharide (LPS)‐induced retinal inflammation [15].

AD16 (GIBH‐130) is a new small molecule that inhibits LPS‐induced neuroinflammation and enhances learning and memory in Alzheimer's disease (AD) mice [16, 17]. In neonatal rat models of brain injury, AD16 significantly improves sensorimotor function and reduces cerebral infarction, likely by lowering pro‐inflammatory signals STAT3 and Nuclear factor‐kappaB (NF‐κB) phosphorylation and activating MAPK/ERK signaling [18]. Does AD16 modulate inflammation via α7nAChR, influencing microglial activation and polarization after transient middle cerebral artery occlusion (tMCAO) model in rats, and thus offer therapeutic benefits for adult CI/RI? This study aims to clarify AD16's therapeutic effects on acute CI/RI and explore its mechanisms, providing a foundation for ischemic stroke drug development.

## 2 Materials and Methods

### 2.1 Animals

Male SD rats (240–280 g, SPF grade) were obtained from Hunan SLAC Laboratory Animal Co. Ltd. and housed at 22°C–25°C with 55%–65% humidity and a 12‐h light/dark cycle, with free access to food and water. The experiment adhered to Gannan Medical University's Ethics Committee regulations.

### 2.2 Drugs and Reagents

AD16 was procured from Shanghai Skychemical Co. Ltd. (Shanghai, China) and dissolved in a 2% dimethyl sulfoxide (DMSO) to 1 mg/mL concentration. Alpha‐bungarotoxin (α‐BTX) was obtained from GLPBIO Corporation (catalog no. GC20230‐1 mg) and dissolved in phosphate buffer solution. U0126 was acquired from Sigma Corporation (catalog no. 109511‐58‐2) and dissolved in DMSO. The primary antibodies used in this study were procured from various suppliers: Iba‐1 (ab5076), CD40 (ab13545), CD68 (ab125212), CD206 (ab125028), CD11b (ab133357), and α7nAChR (ab216485) antibodies were purchased from Abcam; p‐ERK (4370 s), ERK (4695 s), p‐STAT3 (9145 s), and STAT3 (9139 s) antibodies were obtained from Cell Signaling Technology; β‐tubulin (10094–1‐AP), β‐actin (66009‐1‐Ig) were purchased from Proteintech; IL‐6 (WL02841), TNF‐α (WL01581), IL‐10 (WL03088), occludin (WL01996), and AQP4 (WL02267) antibodies were sourced from Wanleibio (Shenyang, China); claudin‐5 (34‐1600) and ZO‐1 (61‐7300) antibodies were acquired from Invitrogen; IL‐1β (AF7209) antibody was sourced from Beyotime Biotechnology Company; and TLR4 (sc‐293072) antibody was purchased from Santa Cruz Biotechnology. Rat IL‐6 ELISA kit was purchased from R&D Systems. Rat IL‐10 and TNF‐α ELISA kits were purchased from Proteintech.

### 2.3 Experimental Design

The experiment consists of two parts. In the first part, rats were randomly assigned to four groups: Sham, AD16, tMCAO, and tMCAO + AD16. Ten minutes post‐ischemia, AD16 (1 mg/kg) was administered via intraperitoneal injection to the AD16 and tMCAO + AD16 groups, while the Sham and tMCAO groups received a solvent. In the second part, rats were divided into three groups: tMCAO + AD16 + Vehicle, tMCAO + AD16 + α‐BTX, and tMCAO + AD16 + U0126. Before tMCAO, the three groups received intracerebroventricular injections of Vehicle, α‐BTX, and U0126, respectively. Following tMCAO, all groups were given an intraperitoneal injection of 1 mg/kg of AD16 [19].

### 2.4 Transient Middle Cerebral Artery Occlusion (tMCAO) Model

The tMCAO model was created using Zea Longa's wire bolt method [20]. Briefly, male SD rats were anesthetized with 4%–5% isoflurane, immobilized, the neck skin was prepared, and the surgical area was disinfected with 75% alcohol. A small incision is performed along the midline of the neck to expose and isolate the right external carotid artery (ECA), internal carotid artery (ICA), and common carotid artery (CCA). A distal incision is then made on the ECA, through which a suture filament is introduced. This filament is advanced to occlude the middle cerebral artery by reaching the anterior cerebral artery for a duration of 120 min, after which it is retracted at the ECA site, and the incision is sutured. In the sham‐operated group, rats undergo exposure and isolation of the CCA, ECA, and ICA without the occlusion of the middle cerebral artery. Throughout the surgical procedure, the room temperature was strictly controlled between 23°C–25°C.

### 2.5 Intracerebroventricular Injections

After anesthetizing the rats, they were secured in a stereotaxic apparatus. The head hair was trimmed, the skin disinfected with 75% alcohol, and the skull exposed via a longitudinal incision. Using the fontanel as a reference point (0.9 mm posterior, 1.6 mm right), holes were drilled with a skull drill. A microinjector was then inserted 3.5 mm subdurally to reach the lateral ventricle. Rats in each group received a 1 μL injection of the respective inhibitor (U0126 at 1 μg/μL, α‐BTX at 1 μg/μL, or Vehicle) into the lateral ventricle at 0.2 μL/min [21, 22]. The needle was left in place for 3 min, partially withdrawn by 3 mm, left for another 3 min, and then fully removed. The skin was sutured afterward.

### 2.6 Tests of Neurological Function

Motor, sensory, and coordination functions were assessed 24 h post‐CI/RI using Garcia JH and Longa scores [23]. The Garcia JH score evaluated the degree of neurological impairment in six aspects, including autonomic movement, forelimb extension function, climbing movement, etc. The highest score was 18 points, and the lowest score was 3 points. A higher score indicates better function. The Longa score ranges from 0 to 4: 0 means normal with no neurological deficits; 1 means the opposite forepaw can notfully extend; 2 means walking in circles towards the surgery side; 3 means leaning towards the surgery side while walking; and 4 means unconscious and unable to walk. A higher score signifies a more severe injury.

### 2.7 Rotarod Test

The Rotarod test assesses rodent motor function [24]. The rod's speed starts at 4 rpm and accelerates to 40 rpm over 5 min, with a 6‐min training period. Rats are positioned with tails outward, preventing head turns, and their fall speed and time on the rod are recorded. Training occurs twice daily for 2 days before tMCAO, with the main experiment starting 1 day post‐surgery.

### 2.8 CatWalk‐Automated Gait Analysis

CatWalk gait analysis system was used to assess gait and motor coordination post‐CI/RI, which is mainly composed of a walking table, camera, image acquisition system, computer, etc. Two days before the operation, rats underwent twice‐daily pre‐training, excluding low performers. To prevent odor and footprints interference, the trail was sprayed with 75% alcohol after testing each rat. During the test, rats walked continuously on a platform for 0.5–8 s, maintaining a speed variation within 60%. Effective running data were collected three times per rat.

### 2.9 TTC Staining

After anesthetizing the rats, the brain was extracted, the cerebellum removed, and the brain divided into five 2 mm thick pieces. These were stained with 5% TTC solution at 37°C for 15 min. Post‐staining, the TTC solution was collected, the tissue washed with PBS, and fixed in 4% paraformaldehyde for 6–8 h. The CanoScan LiDE 300 was used for scanning, and infarction volume (%) was analyzed with ImageJ software using the formula: infarct volume (%) = [(area of contralateral hemisphere − area of non‐infarcted ipsilateral hemisphere)/(2 × area of contralateral hemisphere)] × 100% [19].

### 2.10 Brain Water Content Measurement

After anesthetizing the rats, their brains were severed and the cerebellum removed. The brains were halved, with the right half weighed for its wet weight. The left half was baked at 60°C for 72 h and then weighed for its dry weight. The right brain's water content was calculated using the formula: [(right brain wet weight − left brain dry weight)/right brain wet weight] × 100%.

### 2.11 ELISA

The ischemic cortex was made into a 10% brain tissue homogenate with saline, then centrifuged at 4°C for 10 min at 3500 rpm. The supernatant was collected and analyzed per kit instructions. IL‐6, IL‐10, and TNF‐α levels were measured using ELISA, and protein content was determined by BCA. Cytokine levels were expressed per milligram of protein.

### 2.12 Western Blot

After anesthetizing the rats, ischemic penumbra tissues were collected. Tissue homogenates were prepared and centrifuged at 12,000 rpm at 4°C to measure protein concentration using BCA. Proteins were separated by SDS‐PAGE, transferred to PVDF membranes, and blocked with 5% skim milk for 1 h. Then, the membranes were incubated overnight at 4°C with the following primary antibody solution: Iba‐1 (1/1000), CD11b (1/1000), CD206 (1/1000), CD68 (1/1000), CD40 (1/1000), α7nAChR (1/1000), IL‐1β (1/500), IL‐6 (1/1000), TNF‐α (1/1000), IL‐10 (1/1000), Occludin (1/1000), Claudin‐5 (1/1000), ZO‐1 (1/500), AQP4 (1/1000), ERK (1/1000), p‐ERK (1/1000), STAT3 (1/1000), p‐STAT3 (1/1000), TLR4 (1/1000). Followed by secondary antibodies incubated at room temperature for 1 h, and the membrane was developed using an Amersham Imager 600 system. ImageJ software calculated the gray value of the target protein bands, normalized with the internal reference, and GraphPad Prism 9.0 was used for analysis.

### 2.13 Immunofluorescence Staining

The fixed rat brains were dehydrated in 20% and 30% sucrose solutions, embedded, and sliced into 30 μm sections using a cryomicrotome. Selected sections were washed with PBST, blocked with 3% BSA for 1 h, and incubated overnight at 4°C with primary antibodies Iba‐1, CD206, and CD68 (all at 1:300). After washing with PBST for 3 × 10 min, sections were incubated for 1 h with Alexa Fluor 546 donkey anti‐rabbit IgG (H + L) or Alexa Fluor 488 donkey anti‐goat IgG (H + L). DAPI was added for a 10‐min stain, followed by mounting and baking for 4 h. The sections were then sealed with an antifluorescent quencher, imaged using a Zeiss fluorescence microscope, and analyzed.

### 2.14 Nissl Staining

The brain slices were washed with PBST for 10 min, mounted on slides, and heated at 60°C for 4 h. After three 1‐min PBST washes, 200 μL of Nissl solution was applied to each slice and left at room temperature for 10 min. The slices were then dehydrated in 95% ethanol for 5 s, cleared in xylene for 5 min, sealed, and photographed.

### 2.15 Molecular Docking

The small molecule structures were obtained from the PubChem database (https://pubchem.ncbi.nlm.nih.gov/) and converted to PDB format using OpenBabel 3.1.1. The 3D structure of the protein was retrieved from the Protein Data Bank (https://www.rcsb.org/). PyMOL 2.5.4 was utilized to remove water molecules and ligands from the protein structure. Subsequently, AutoDock Tools 1.5.7 was employed to convert the PDB files of both small molecules and proteins into PDBQT format. Molecular docking was performed using AutoDock Vina 1.1.2, and the optimal docking results were visualized with PyMOL 2.5.4 software.

### 2.16 Cellular Thermal Shift Assay

Human microglia cell (HMC3) (Servicebio, Wuhan, China) were cultured in Dulbecco's modified Eagle medium/High glucose supplemented with 10% (v/v) fetal bovine serum (FBS, Gibco, USA), 100 U/mL penicillin, and 100 U/mL streptomycin at 37°C in 5% CO2 humidified air. The harvested cell suspensions were subjected to three cycles of freeze–thaw using liquid nitrogen, followed by centrifugation at 15,000 × rpm for 20 min at 4°C. The supernatant obtained post‐centrifugation was divided into two aliquots: one was treated with AD16, while the other received an equivalent volume of DMSO. These aliquots were incubated for 2 h at ambient temperature. Subsequently, each lysate was further partitioned into 10 aliquots, which were exposed to heating at various temperatures for 3 min each, followed by a cooling period of 3 min at room temperature. To remove cell debris, the lysates underwent centrifugation at 15,000 × rpm for 15 min at 4°C. The resulting supernatants were then boiled with the addition of 5 × loading buffer and subsequently analyzed via western blotting.

### 2.17 Statistical Analysis

Data processing and statistical analysis of the experimental results were conducted utilizing GraphPad Prism 9.0 software. The statistical outcomes were represented as means ± standard deviations. The normality of the distribution was evaluated using the Shapiro–Wilk normality test (*n* < 50). For normally distributed data, comparisons were made using Student's *t*‐test or one‐way ANOVA by Dunnett's multiple comparisons test. For non‐normally distributed data, the Mann–Whitney *U*‐test and Kruskal‐Wallis test were used to compare two or more groups, respectively. A *p*‐value of less than 0.05 was considered indicative of statistical significance.

## 3 Results

### 3.1 The Protective Effects of AD16 on the Brain During the Acute Phase of tMCAO in Rats

**FIGURE 1:**
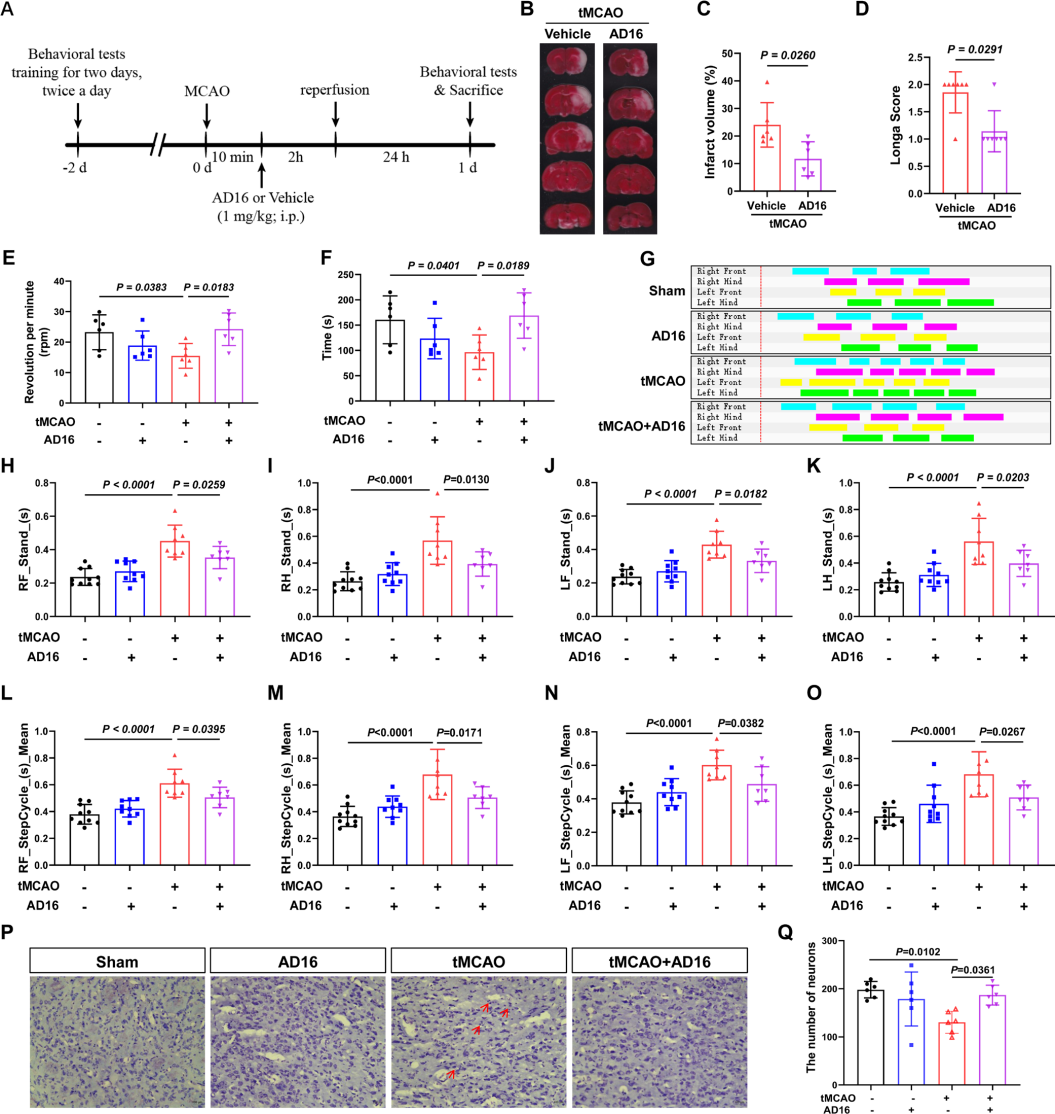
Protective effect of AD16 on brain in acute phase of tMCAO rats. (A) Experimental timeline. (B, C) TTC staining results of each group. (D) Analysis results of Longa score in each group. (E, F) Analysis results of the time on rotarod and the rotating speed of each group. (G) Gait test 2D images of rats standing on their limbs. (H–K) Gait test the support phase of the rat's limbs. (L–O) Gait test the gait cycle of the limbs of rats. (P, Q) The number and morphological changes of Nissl bodies in ischemic cortex of rats were observed by Nissl staining. All data were expressed as Mean ± SD. Panels (C, D) were analyzed by Mann–Whitney *U*‐test. Others were analyzed by one‐way ANOVA following Dunnett's multiple comparisons test. *p* < 0.05 regarded as a statistically significant difference, *n* = 6–10.

To evaluate the impact of AD16 on CI/RI, TTC staining was employed to measure cerebral infarction volume 24 h post‐CI/RI, and neurological function was assessed using Longa score in a double‐blind manner. The experimental results indicated that AD16 treatment significantly reduced cerebral infarction volume and enhanced neural function in tMCAO rats (Figure 1B–D). Additionally, the effects of AD16 on motor function in tMCAO rats were assessed using CatWalk gait analysis and the rotarod test. The experimental results indicated that tMCAO rats had significantly reduced rotarod time and speed compared to the Sham group. AD16 treatment notably improved these metrics (Figure 1E,F). CatWalk gait analysis revealed that tMCAO rats had increased limb stand time and step cycle, which were significantly reduced by AD16 treatment (Figure 1G–O). Furthermore, the impact of AD16 on neuronal survival in tMCAO rats was assessed using Nissl staining. The findings revealed a reduction in the number of Nissl bodies, lighter staining, and neuronal necrosis in the tMCAO group. Conversely, following AD16 treatment, there was an increase in the number of Nissl bodies and more intense staining (Figure 1P,Q). These results suggest that AD16 may confer neuroprotection to the brains of tMCAO rats during the acute stage.

### 3.2 AD16 Enhances the Integrity of the Blood–Brain Barrier and Mitigates Neuroinflammation in tMCAO Rats

**FIGURE 2:**
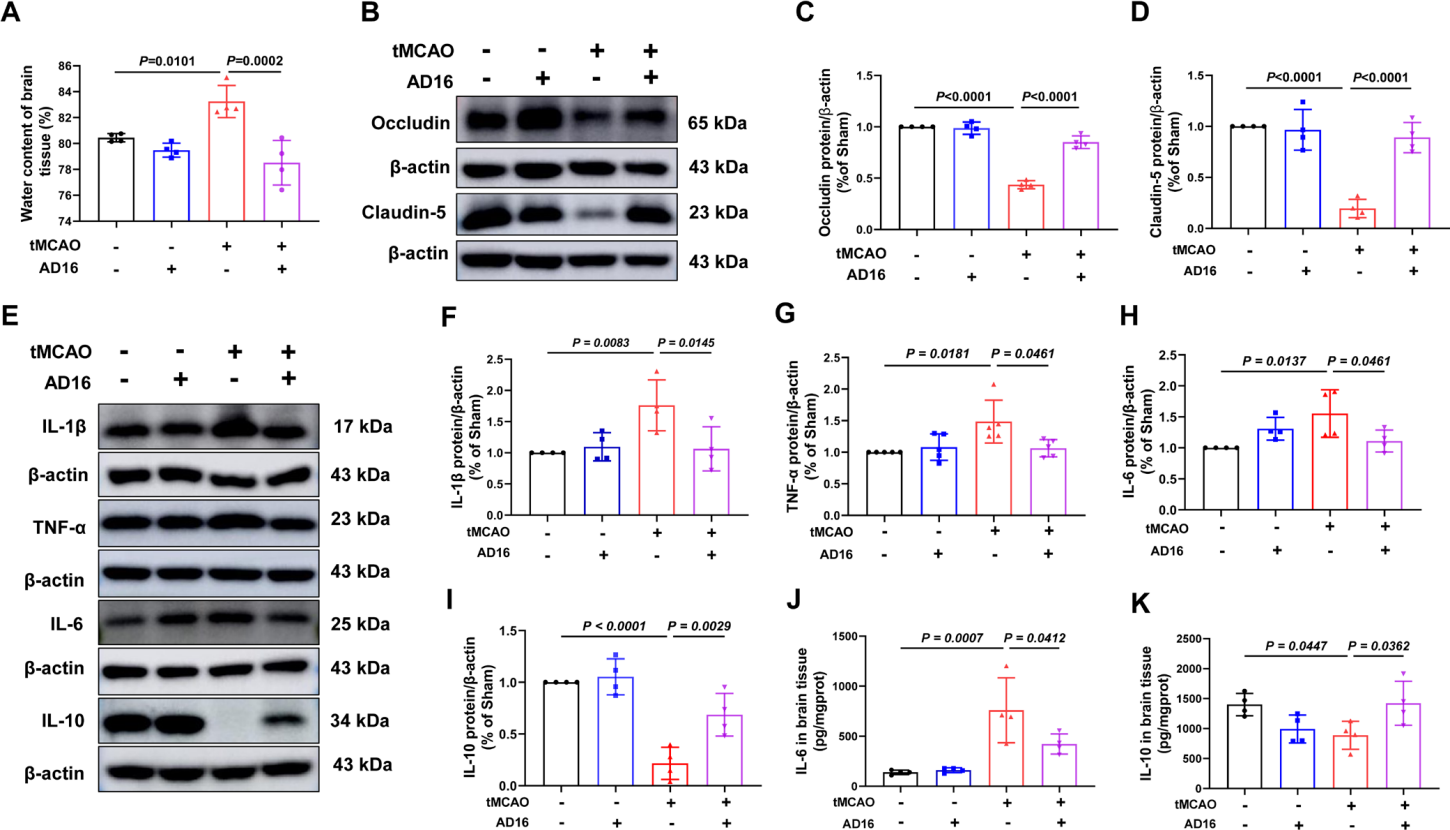
AD16 maintained BBB integrity and reduced neuroinflammation in tMCAO rats. (A) The water content in the right brain of rats. (B) The representative graph of Western blot results of Occludin and Claudin‐5 proteins. (C, D) The statistical graph of Western blot results of Occludin and Claudin‐5 proteins. (E) The representative graph of Western blot results of IL‐1β, TNF‐α, IL‐6, IL‐10 proteins. (F–I) The statistical graph of Western blot results of IL‐1β, TNF‐α, IL‐6, IL‐10 proteins. (J, K) ELISA results of IL‐6 and IL‐10 proteins. All data were expressed as Mean ± SD and were analyzed by one‐way ANOVA following Dunnett's multiple comparisons test (A–D, H–K) or Kruskal‐Wallis test (F, G), with *p* < 0.05 regarded as a statistically significant difference, *n* = 4.

Following an ischemic stroke, the blood–brain barrier (BBB) is compromised, allowing white blood cells to infiltrate the brain and cause inflammation and edema [25]. To assess AD16's impact on BBB integrity, we measured brain water content and tight junction proteins. 24 h post‐CI/RI, the tMCAO group showed a significant increase in brain water content compared to the Sham group, which was notably reduced with AD16 treatment (Figure 2A). In the tMCAO group, tight junction protein claudin‐5 and occludin levels dropped significantly but rose notably after AD16 treatment (Figure 2B–D). In order to explore AD16's impact on neuroinflammation in tMCAO rats, we detected pro‐inflammatory factors IL‐1β, TNF‐α, IL‐6 and anti‐inflammatory factor IL‐10 expression in the ischemic penumbra cortex. Compared to the Sham group, tMCAO group showed a significant increase in IL‐1β, TNF‐α and IL‐6, and a significant decrease in IL‐10, while AD16 treatment could effectively reverse these inflammatory factors expression after CI/RI (Figure 2E–K). These results suggest that AD16 can improve BBB integrity and reduce neuroinflammation in tMCAO rats.

### 3.3 The Impact of AD16 on the Activation and Polarization of Microglia in tMCAO Rats

**FIGURE 3:**
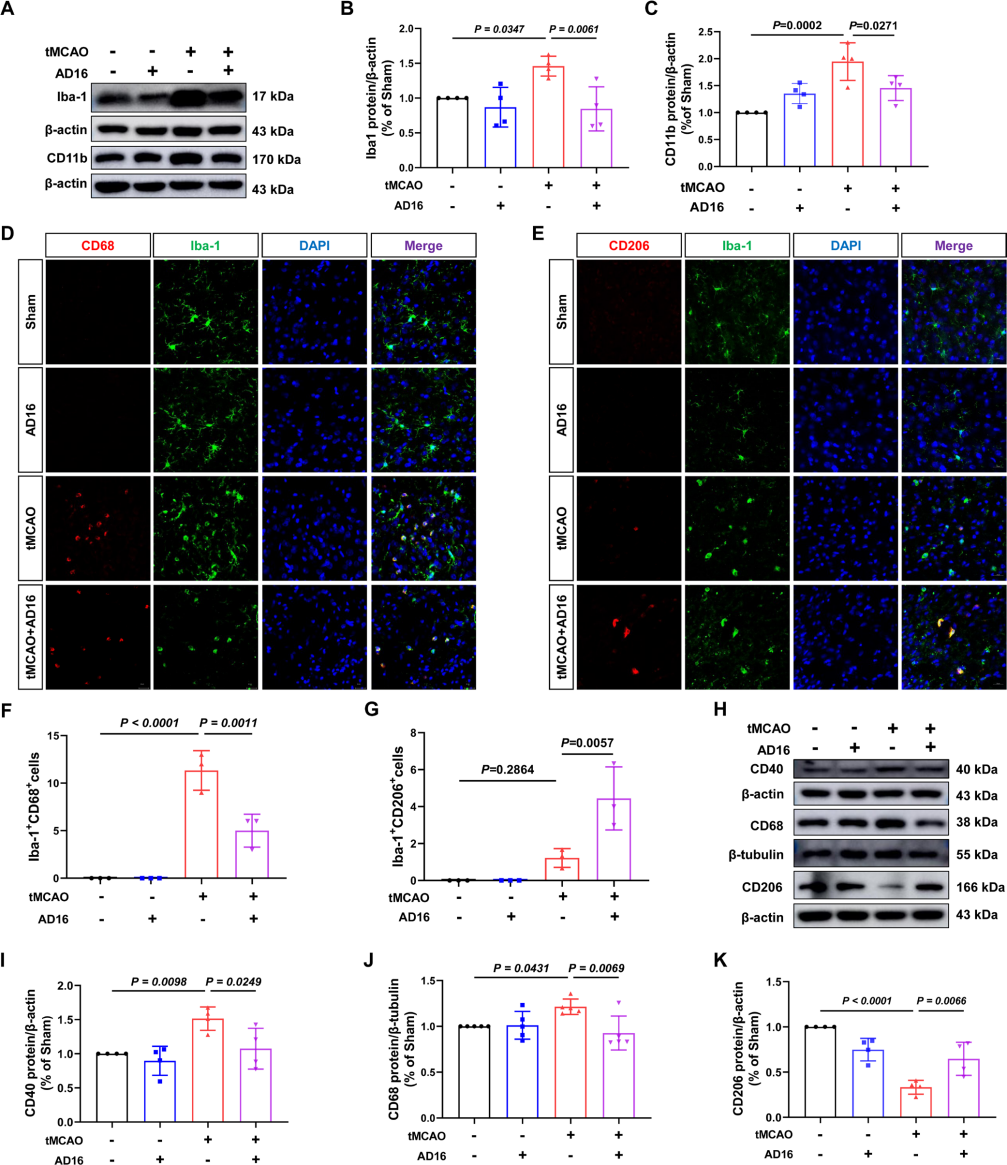
Effect of AD16 on activation and polarization of microglia in tMCAO rats. (A) The representative graph of Western blot results of Iba‐1 and CD11b proteins. (B, C) The statistical graph of Western blot results of Iba‐1 and CD11b proteins. (D, E) The representative graph of immunofluorescence of M1 and M2 microglia. Microglia were labeled with Iba‐1 (green), M1 microglia with CD68 (red), M2 microglia with CD206 (red), Scale bar: 20 μm. (F) The statistical graph of Iba‐1^+^CD68^+^ cells. (G) The statistical graph of Iba‐1^+^CD206^+^ cell. (H) The representative graph of Western blot results of CD40, CD68, CD206 proteins. (I–K) The statistical graph of Western blot results of CD40, CD68 and CD206 proteins. All data were expressed as Mean ± SD and analyzed by one‐way ANOVA following Dunnett's multiple comparisons test, with *p* < 0.05 regarded as a statistically significant difference, *n* = 3–4.

During the acute phase of CI/RI, activated microglia release pro‐inflammatory cytokines and compromise the BBB. To assess whether AD16 reduces neuroinflammation by influencing microglia activation and polarization, we analyzed Iba‐1 and CD11b (markers of microglia activation) expression in the ischemic penumbra of tMCAO rats. Compared to the Sham group, the tMCAO group showed an increase in Iba‐1 positive cells and higher protein levels of Iba‐1 and CD11b, while both were significantly decreased after AD16 treatment (Figure 3A–C,D). Subsequently, we assessed CD68 and CD40 (M1 microglia markers) and CD206 (M2 microglia marker) via immunofluorescence staining and Western blot to determine microglia polarization. In comparison to the Sham group, the tMCAO group demonstrated increased co‐localization of Iba‐1 and CD68, along with elevated protein levels of CD68 and CD40. These increases were significantly attenuated following AD16 treatment (Figure 3D,F,H–J). Furthermore, while the tMCAO group exhibited a trend towards a higher number of cells co‐localized with Iba‐1 and CD206, this difference did not reach statistical significance. The protein level of CD206 was reduced in the tMCAO group; however, a significant increase was observed post‐AD16 treatment (Figure 3E,G,H,K). These findings suggest that AD16 may inhibit microglial activation and M1‐type polarization while promoting M2‐type polarization.

### 3.4 The Influence of AD16 on the α7nAChR‐ERK‐STAT3 Signaling Pathway

**FIGURE 4:**
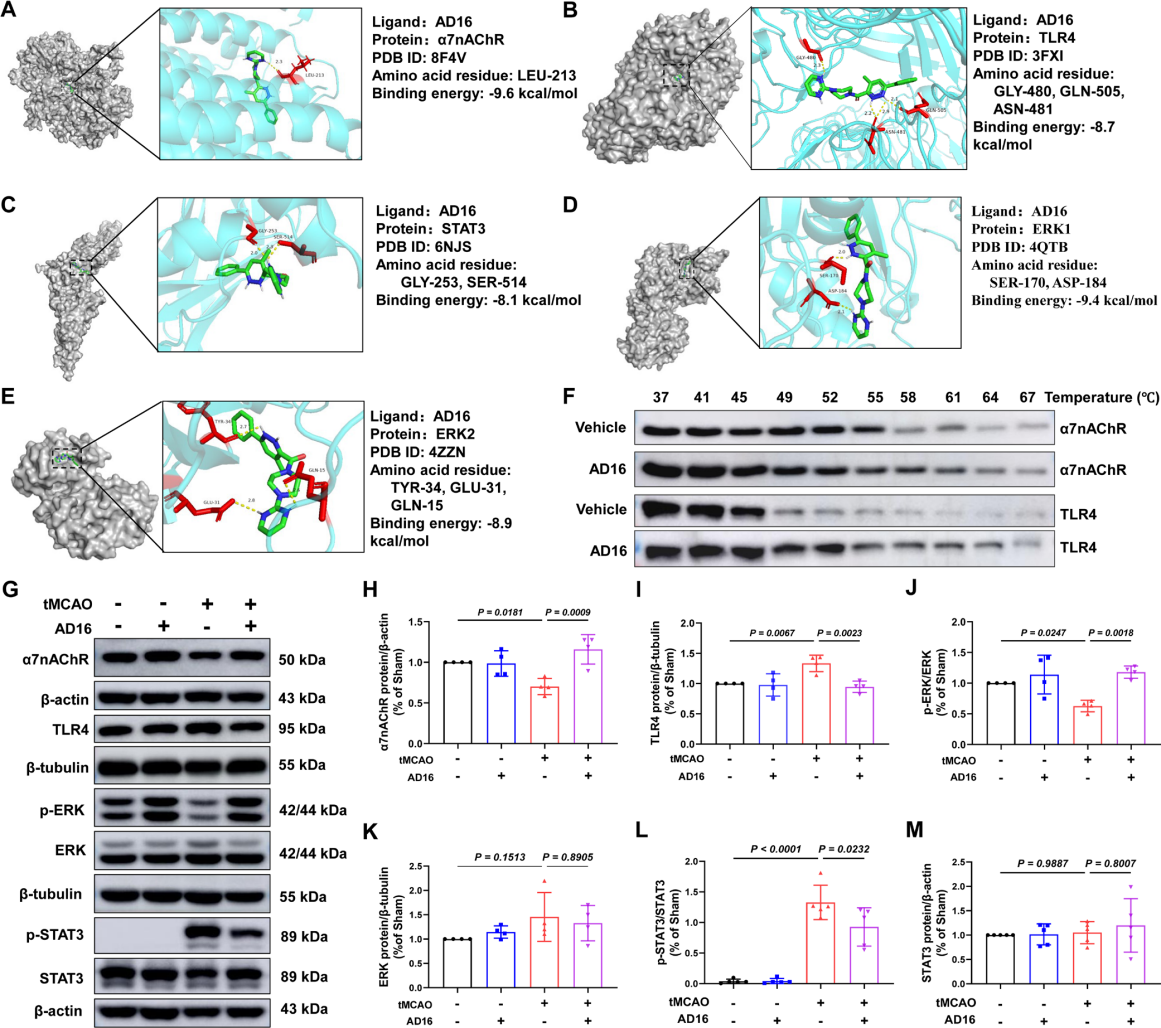
Effect of AD16 on α7nAChR‐ERK‐STAT3 signaling pathway. (A–E) Molecular docking of α7nAChR, TLR4, STAT3, ERK1, and ERK2 with AD16. (F) Cellular thermal shift assay (CETSA) analysis indicated the α7nAChR andTLR4 degradation at different temperatures with or without AD16 treatment. (G)The representative graph of Western blot results of α7nAChR, TLR4, ERK, and STAT3 and their phosphorylated proteins. (H–M) The statistical graph of Western blot results of α7nAChR, TLR4, ERK, STAT3, and their phosphorylated proteins. All data were expressed as Mean ± SD and analyzed by one‐way ANOVA following Dunnett's multiple comparisons test, with *p* < 0.05 regarded as a statistically significant difference, *n* = 4.

α7nAChR, as a key protein in cholinergic anti‐inflammatory pathway, can effectively reduce inflammation [7]. To investigate the binding interaction between AD16 and α7nAChR, we employed molecular docking techniques to simulate the binding pattern and affinity of AD16 to α7nAChR, as well as other downstream signals molecules ERK and STAT3. Typically, a lower binding energy between the receptor and ligand indicates a more stable interaction, with binding energies below −7 kcal/mol generally considered indicative of strong binding affinity [26]. Our findings reveal that AD16 forms a hydrogen bond with the LEU‐213 residue of α7nAChR, with a binding energy of −9.6 kcal/mol. The binding energies of AD16 to ERK1, ERK2, TLR4 and STAT3 are −9.4, −8.9, −8.7 and −8.1 kcal/mol, respectively, all forming hydrogen bonds. This suggests AD16 binds well to α7nAChR, TLR4, ERK1/2, and STAT3 (Figure 4A–E). To further validate whether AD16 genuinely interacts with two critical membrane proteins involved in neuroinflammatory regulation, we employed the Cellular Thermal Shift Assay (CETSA) to evaluate its effects on the thermal stability of α7nAChR and TLR4 at the cellular level. Consistent with the molecular docking results, AD16 treatment significantly delayed the degradation of both proteins, indicating a direct stabilization effect (Figure 4F). Meanwhile, AD16's impact on α7nAChR‐ERK‐STAT3 signaling, were measured by Western blot. The results indicated a significant reduction in α7nAChR and p‐ERK protein levels and an increase in p‐STAT3 protein levels in the tMCAO group compared to the Sham group. These changes were reversed with AD16 treatment (Figure 4G,H,J,L). TLR4 is essential for microglia to control brain inflammation by interacting with various ligands [27]. We investigated if AD16 treatment affects TLR4 protein expression. Our findings revealed that TLR4 levels were elevated in the tMCAO group compared to the Sham group but significantly reduced following AD16 treatment (Figure 4G,I). This indicates that AD16 might regulate α7nAChR‐ERK‐STAT3 and TLR4 signaling pathways.

### 3.5 The Brain‐Protective Effect of AD16 in tMCAO Rats Is Recersede by α‐BTX and U0126

**FIGURE 5:**
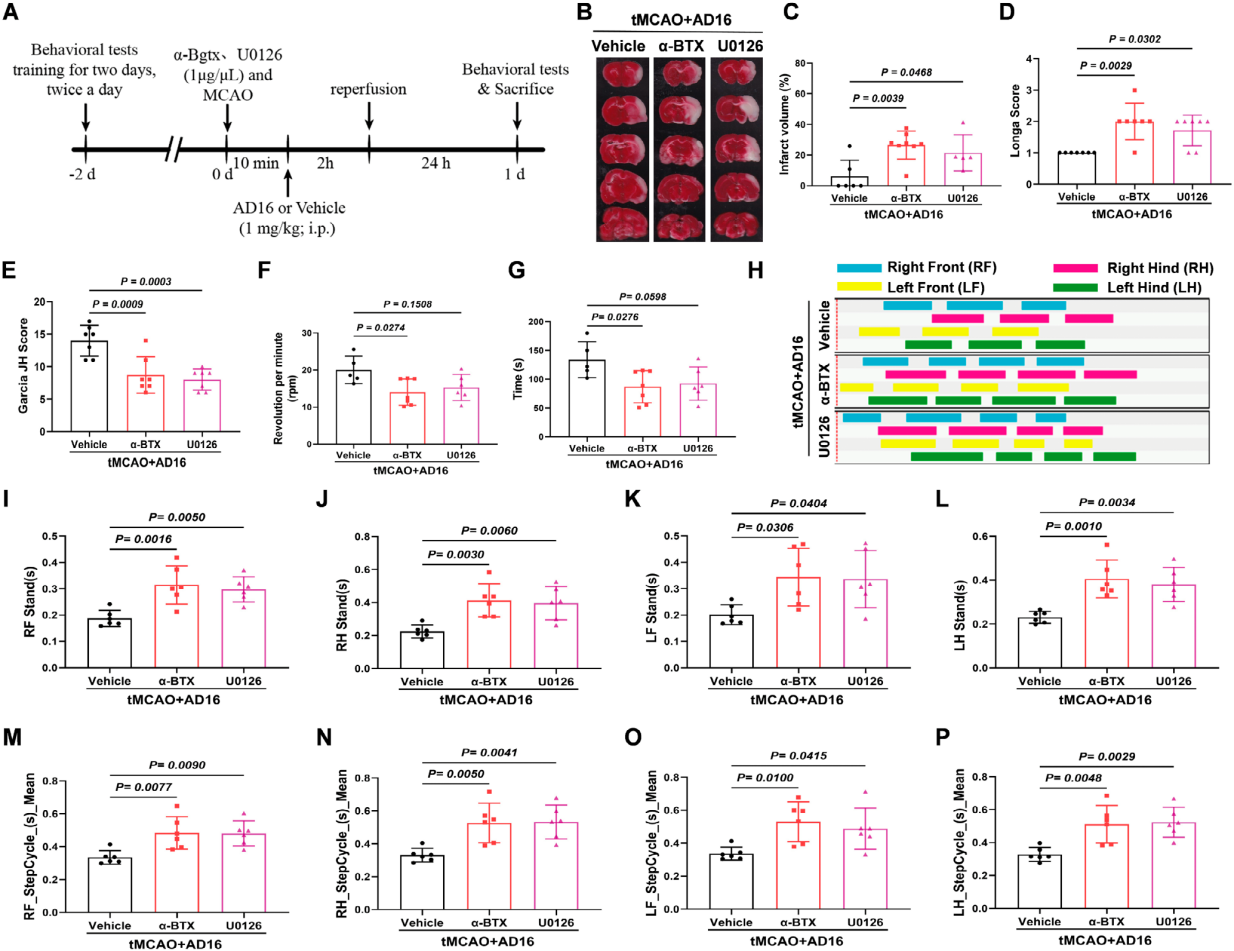
α‐BTX and U0126 reversed the brain protective effect of AD16 on tMCAO rats. (A) Experimental timeline. (B, C) TTC staining results of each group. (D, E) Analysis results of Longa and Garcia JH scores of each group. (F, G) Analysis results of the time on rotarod and the rotating speed of each group. (H) Gait test 2D images of rats standing on their limbs. (I‐L) Gait test of the support phase of the rat's limbs. (M–P) Gait test of the gait cycle of the limbs of rats. All data were expressed as Mean ± SD and were analyzed by one‐way ANOVA following Dunnett's multiple comparisons test (C, E, G–O) or Kruskal‐Wallis test (D, F), with *p* < 0.05 regarded as a statistically significant difference, *n* = 5–8.

To elucidate the roles of α7nAChR and ERK in the regulation of microglial activation and polarization by AD16, we administered α‐BTX (an α7nAChR inhibitor) and U0126 (an ERK inhibitor) into the lateral ventricle of rats via stereotaxic injection. This approach allowed us to observe the effects of these inhibitors on the neuroprotective properties of AD16 (Figure 5A). The results indicated that, in comparison to the Vehicle group, the cerebral infarction area and Longa score were elevated in the α‐BTX and U0126 intervention groups, while the Garcia JH score was reduced. Additionally, there was a significant decrease in both the time spent on the rotarod and the rotating speed of the rats. Conversely, both the stand time and step cycle of the limbs were markedly increased (Figure 5B–P). These findings suggest that the α7nAChR‐ERK signaling pathway may play a crucial role in the neuroprotective effects of AD16.

### 3.6 α‐BTX and U0126 Negate the BBB Protective and Anti‐Inflammatory Effects of AD16

**FIGURE 6:**
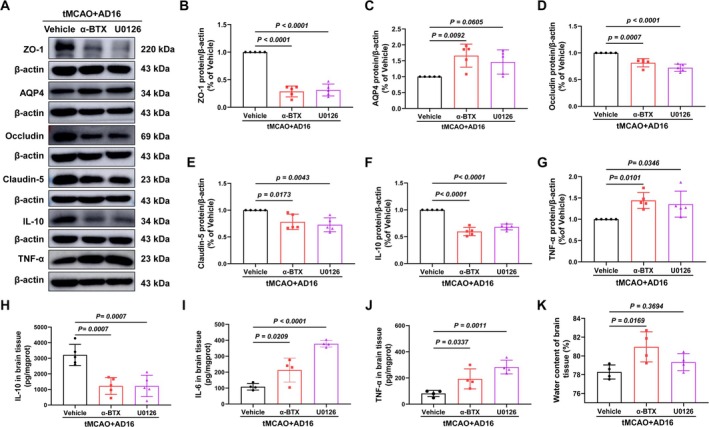
α‐BTX and U0126 reversed the anti‐inflammatory effect of AD16 on tMCAO rats and the protective effect of BBB. (A) The representative graph of Western blot results of ZO‐1, AQP4, Occludin, Claudin‐5, IL‐10, TNF‐α proteins. (B–G) The statistical graph of Western blot results of ZO‐1, AQP4, Occludin, Claudin‐5, IL‐10, TNF‐α proteins. (H–J) ELISA results of IL‐10, IL‐6, TNF‐α proteins. (K) The water content in the right brain of rats. All data were expressed as Mean ± SD and analyzed by one‐way ANOVA following Dunnett's multiple comparisons test, with *p* < 0.05 regarded as a statistically significant difference, *n* = 4–5.

To investigate whether α‐BTX and U0126 could reverse the BBB protective effects of AD16. Compared to the Vehicle group, the levels of BBB tight junction proteins ZO‐1, Occludin, and Claudin‐5 were significantly reduced in the α‐BTX and U0126 intervention groups. Additionally, the α‐BTX treatment group exhibited a significant increase in aquaporin AQP4 expression and brain water content. In the U0126 treatment group, there was an observed increasing trend in these parameters; however, the changes were not statistically significant (Figure 6A–E,K). These findings suggest that AD16 may exert its BBB protective effects through the α7nAChR and ERK pathways.

The pro‐inflammatory factors IL‐6 and TNF‐α, as well as the anti‐inflammatory factor IL‐10, were quantified using ELISA and Western blot assays 24 h following CI/RI. Compared to the Vehicle group, the levels of IL‐6 and TNF‐α were significantly elevated in the α‐BTX and U0126 groups, whereas the protein levels of IL‐10 were significantly reduced (Figure 6A,F–J). These findings indicate that AD16 may mitigate neuroinflammation via modulation of the α7nAChR and ERK pathway.

### 3.7 α‐BTX and U0126 Counteract the Regulatory Effects of AD16 on Microglia Activation and Polarization

**FIGURE 7:**
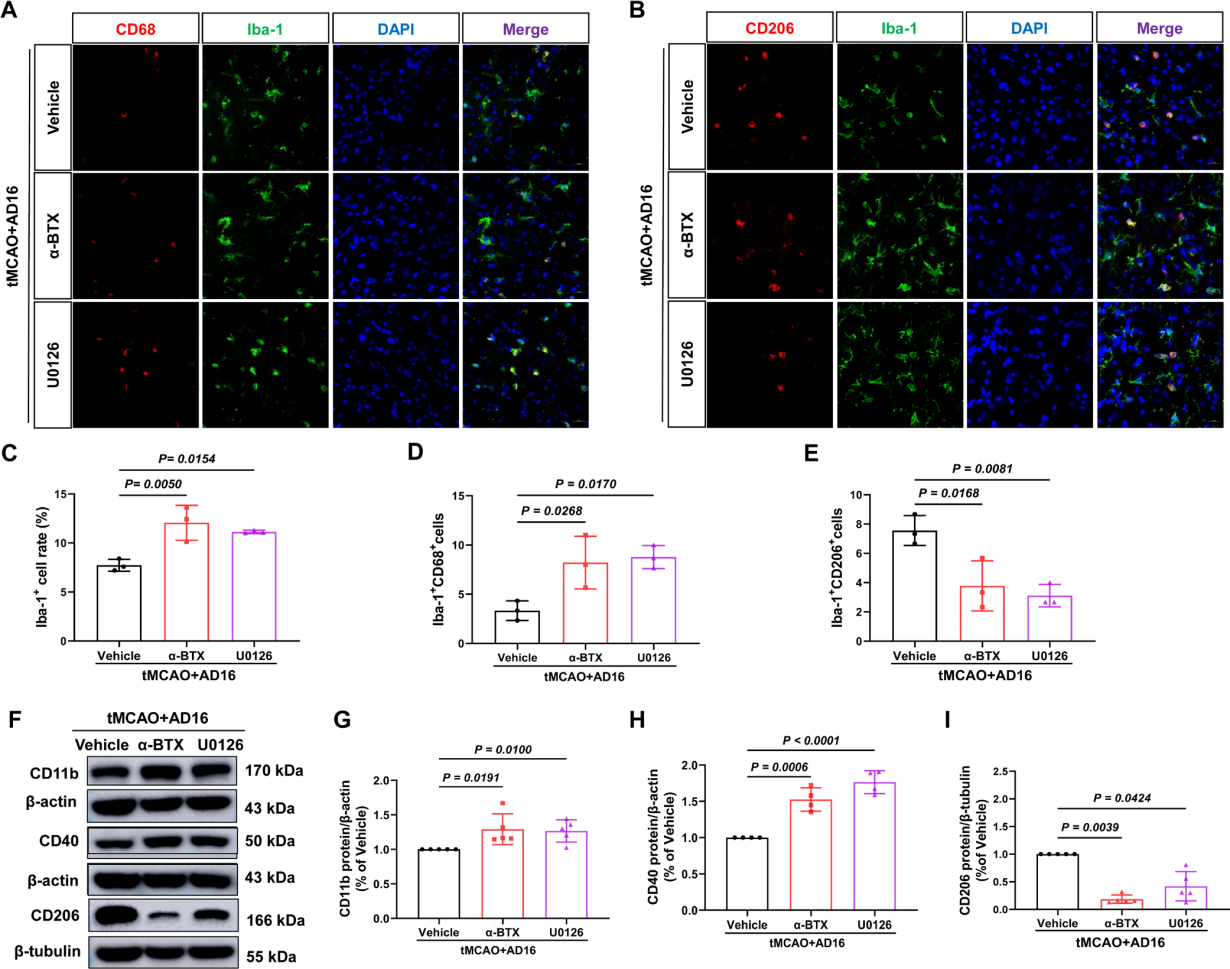
α‐BTX and U0126 reversed the regulatory effect of AD16 on the activation and polarization of microglia in tMCAO rats. (A, B) The representative graph of immunofluorescence of M1 and M2 microglia, Scale bar: 20 μm. (C) Microglia positive cell rate. (D) The statistical graph of Iba‐1^+^CD68^+^ cells. (E) The statistical graph of Iba‐1^+^CD206^+^ cells. (F) The representative graph of Western blot results of CD11b, CD40, and CD206 proteins. (G–I) The statistical graph of Western blot results of CD11b, CD40, and CD206 proteins. All data expressed as Mean ± SD were analyzed by one‐way ANOVA following Dunnett's multiple comparisons test (C–E, H) or Kruskal‐Wallis test (G, I), with *p* < 0.05 regarded as a statistically significant difference, *n* = 3–5.

Subsequently, we investigated whether interventions with α‐BTX and U0126 could reverse the regulatory effects of AD16 on microglial activation and polarization within the ischemic penumbra of tMCAO rats, utilizing immunofluorescence and Western blot analyses. Immunofluorescence results indicated that, in comparison to the Vehicle group, both α‐BTX and U0126 interventions led to an increase in the rate of microglia‐positive cells and the number of M1 microglia, while concurrently reducing the number of M2 microglia (Figure 7A–E). Western blot analysis revealed that α‐BTX and U0126 treatment raised CD11b and CD40 protein levels and reduced CD206 levels compared to the Vehicle group (Figure 7F–I). This indicates that α7nAChR and ERK might play a role in AD16's regulation of microglial activation and polarization.

### 3.8 α‐BTX and U0126 Reversed the Phosphorylation of ERK and STAT3 by AD16

**FIGURE 8:**
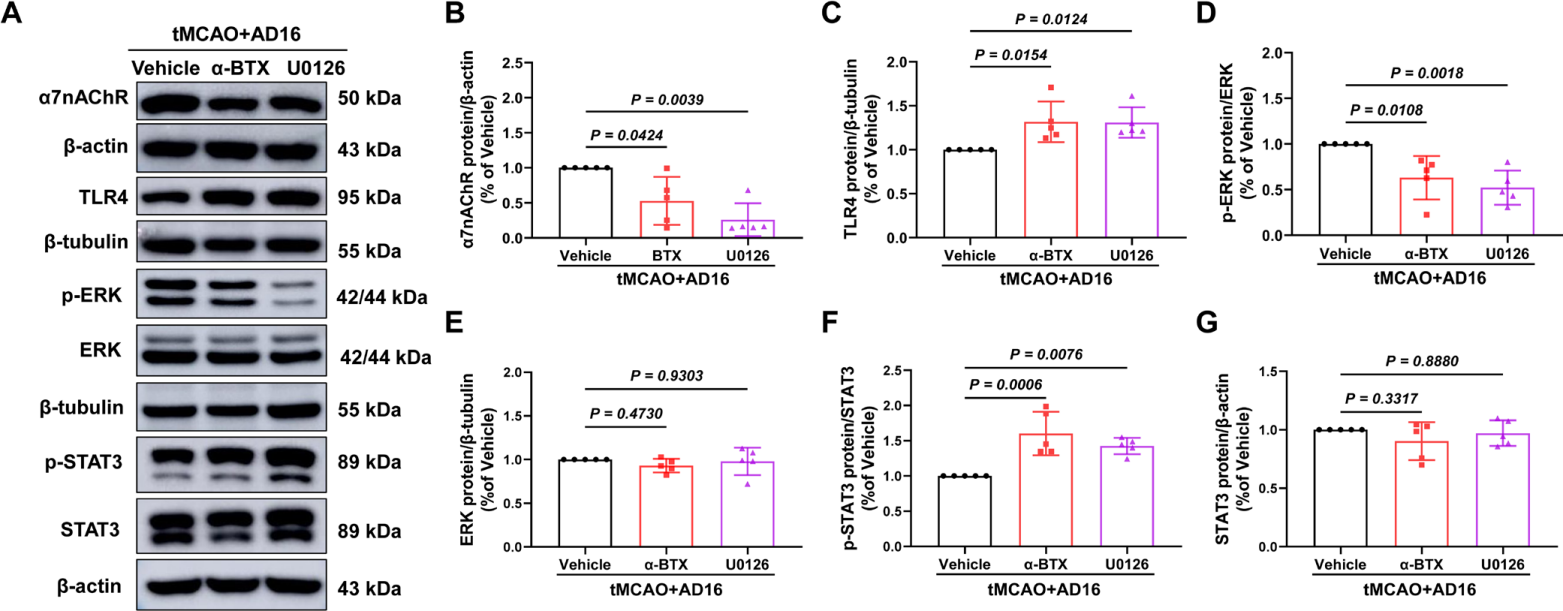
α‐BTX and U0126 reversed the phosphorylation of AD16 on ERK and STAT3. (A) The representative graph of Western blot results of α7nAChR, TLR4, ERK, STAT3, and their phosphorylated proteins. (B–G) The statistical graph of Western blot results of α7nAChR, TLR4, ERK, STAT3, and their phosphorylated proteins. All data were expressed as Mean ± SD and were analyzed by one‐way ANOVA following Dunnett's multiple comparisons test (D–G) or Kruskal‐Wallis test (B, C), with *p* < 0.05 regarded as a statistically significant difference, *n* = 5.

Compared to the Vehicle group, interventions with α‐BTX and U0126 modified the up‐regulatory effect of AD16 on α7nAChR protein and the down‐regulatory effect of AD16 on TLR4 protein. This led to a reduction in p‐ERK levels and an elevation in p‐STAT3 levels, without significant alterations in total protein levels (Figure 8A–G). These findings indicate that AD16 may inhibit the phosphorylation of STAT3 through the interaction between α7nAChR and ERK.

## 4 Discussion

AD16 is a promising candidate drug for the treatment of Alzheimer's disease, primarily by inhibiting neuroinflammation to improve the pathological cascade of AD [28]. AD16 offers several advantages over other anti‐neuroinflammatory drugs: it easily crosses the BBB, has a high oral bioavailability of 74.91%, a long half‐life of 4.32 h, and shows no significant toxic side effects even with continuous high‐dose (250 mg/kg) oral administration [17]. Our previous studies have shown that AD16 reduces the cerebral infarct volume and improve neurological function in tMCAO rats, with the most significant therapeutic effect observed at a dose of 1 mg/kg [19]. However, it remains unclear if AD16's brain‐protective effects involve regulating microglia activation and polarization, key players in neuroinflammation during CI/RI. Therefore, this study employs this dosage to explore its therapeutic effects and molecular mechanisms in tMCAO rats during the acute phase from the perspective of microglia. Additionally, we found that after AD16 treatment, the number of Nissl bodies in the ischemic cortical side of tMCAO rats increased, rescuing neuronal death.

The BBB consists of endothelial cells interacting with pericytes, astrocytes, neurons, and microglia in neurovascular units [29, 30]. Ischemic stroke often leads to BBB damage, marked by decreased tight junction proteins and structural changes [31]. This allows immune cells and plasma components to infiltrate the brain, worsening inflammation and edema [32]. Additionally, CI/RI activates brain microglia and astrocytes, increasing inflammatory factors and chemokines, thus intensifying neuroinflammation [25]. BBB injury and neuroinflammation are interrelated and together contribute to CI/RI. Activation of nuclear factor E2 related factor 2 (NRF2) can protect BBB integrity by inhibiting the inflammatory response [33]. Our study found that AD16 enhances tight junction proteins Occludin and Claudin‐5, lowers brain water content and pro‐inflammatory cytokines IL‐1β, IL‐6, and TNF‐α, while increasing the anti‐inflammatory factor IL‐10 in tMCAO rats. This indicates that AD16 may protect the brain by reducing neuroinflammation and BBB injury.

Microglia, constituting 10%–15% of central nervous system cells, serve as the primary innate immune responders within the brain parenchyma [4, 34]. Their activation in the context of cerebral ischemia exhibits a dualistic nature, attributable to the presence of two distinct polarization states: the anti‐inflammatory and pro‐inflammatory phenotypes [35]. The present study demonstrates that AD16 effectively inhibits microglial activation in tMCAO rats. Simultaneously, we investigated the impact of AD16 on the M1/M2 phenotypic polarization of microglia. Our findings indicate that AD16 reduces the expression of M1 microglia markers while enhancing the expression of M2 microglia markers. These results imply that AD16 mitigates neuroinflammation induced by transient middle cerebral artery occlusion (tMCAO) by promoting M2‐type polarization and inhibiting M1‐type polarization of microglia.

CAP is crucial in ischemic stroke. Studies indicate that puerarin treatment in tMCAO rats decreases cerebral infarction volume, enhances neural function, and lowers pro‐inflammatory factors IL‐1β, IL‐6, and TNF‐α, likely via CAP activation [36]. The α7nAChR, a key regulator of the brain's cholinergic anti‐inflammatory pathway, is found in astrocytes and microglia. Activating α7nAChR in microglia reduces pro‐inflammatory factors [37, 38]. In vitro studies have indicated that activation of the α7nAChR can inhibit LPS‐induced M1‐type microglial polarization while promoting M2‐type microglial polarization [39]. Additionally, α‐BTX has been demonstrated to activate NF‐κB signaling, thereby promoting the activation of microglia and subsequently increasing the release of pro‐inflammatory cytokines IL‐1β and IL‐6 [40]. Our findings indicate that AD16 effectively binds to α7nAChR, upregulates its expression, and its effects on tMCAO rats are negated by α‐BTX, implying α7nAChR as a key target of AD16.

ERK, a key MAPK family member involved in cell signaling, promotes M2 microglia polarization and offers neuroprotection in rats with chronic cerebral hypoperfusion [41, 42]. Our study showed that AD16 can promote the phosphorylation of ERK, and U0126 reversed the regulatory effect of AD16 on activation and polarization of microglia. The transcription factor STAT3 also plays a key role in regulating neuroinflammation. Research indicates that inhibiting STAT3 activation could be a therapeutic target post‐stroke [43]. After cerebral ischemia, STAT3 can be activated, and then promote the transcriptional regulation of several key molecules inducing brain injury. In mice subjected to middle cerebral artery occlusion (MCAO), the inhibition of STAT3 phosphorylation via JAK2 inhibitors has been demonstrated to suppress the expression of pro‐inflammatory cytokines [44]. Our research findings indicate that AD16 enhances ERK activation while concurrently inhibiting STAT3 activation, an effect that is reversed by α‐BTX. These observations suggest that the α7nAChR may exert an anti‐inflammatory role through the modulation of ERK‐STAT3 signaling pathways. Previous research has demonstrated that low‐dose ketamine can inhibit neuronal apoptosis and neuroinflammation by modulating the TLR4/MAPK/NF‐κB signaling pathway via α7nAChR [45]. In the present study, we observed that AD16 decreased the expression of TLR4, an effect that was reversible upon administration of α‐BTX and U0126. We hypothesized that α7nAChR and ERK interact to regulate inflammation‐related signals (TLR4/MAPK/NF‐κB and STAT3), reducing neuroinflammation. However, TLR4 was not examined in this study, requiring further verification.

Several limitations in this study should be mentioned, including the unresolved binding mechanism of AD16 to α7nAChR and the potential for α‐BTX and U0126 inhibitors to affect targets other than α7nAChR or ERK. To further elucidate the regulatory effects of AD16 on microglial activation and polarization, as well as the molecular mechanisms underlying its inhibition of neuroinflammation, future studies should employ gene knockout techniques to specifically target and ablate the α7nAChR protein in microglia. This approach will help determine whether the observed effects of AD16 are influenced by the presence of α7nAChR. Such investigations are essential for clarifying the cellular and molecular mechanisms through which AD16 inhibits neuroinflammation and contributes to the treatment of ischemic stroke.

**FIGURE 9:**
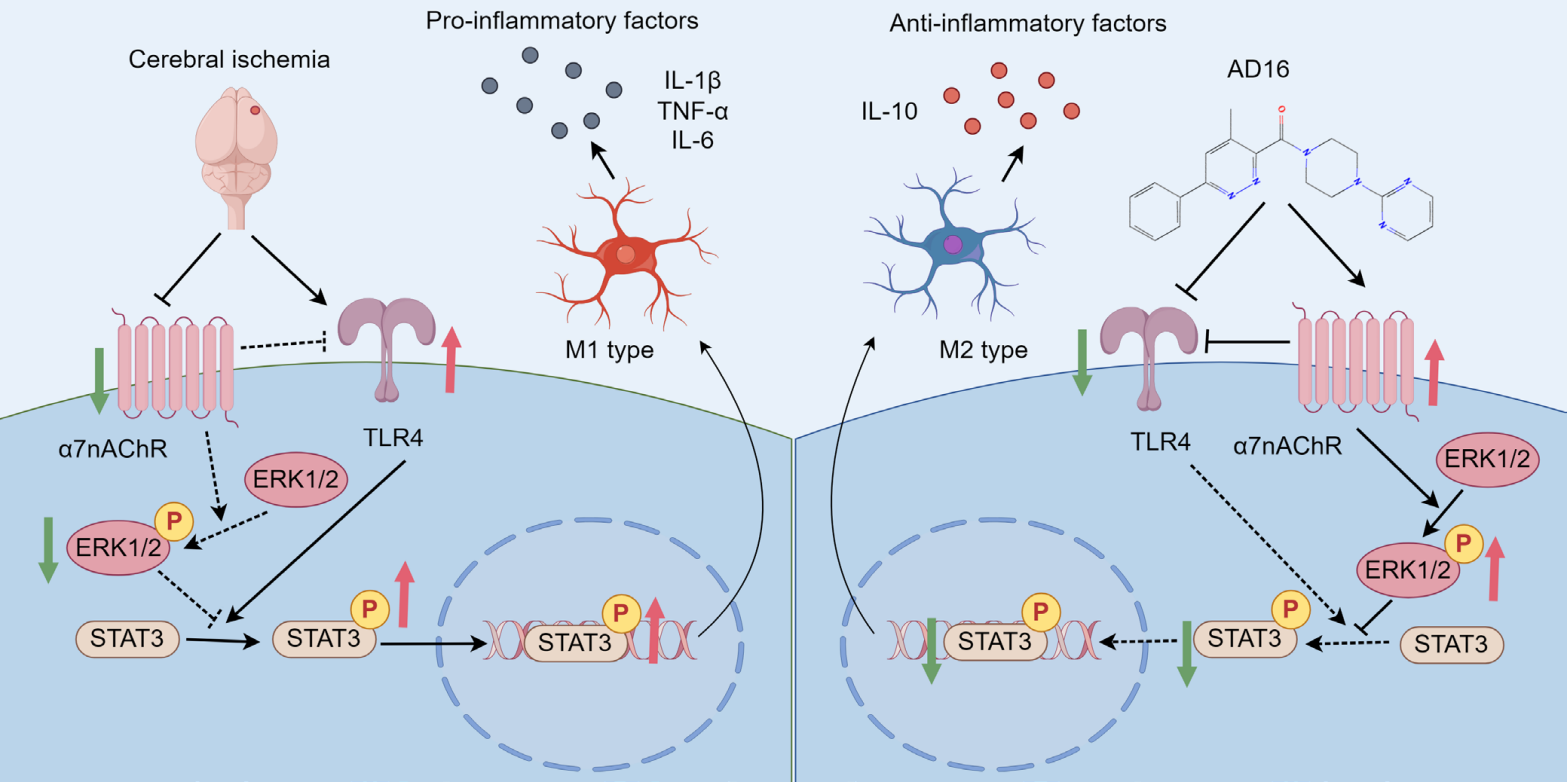
AD16 regulates M1/M2 microglia polarization by α7nAChR‐ERK‐STAT3 signaling to restrain neuroinflammation induced by ischemic stroke.

In summary, our experimental results demonstrated that AD16 inhibited the acute inflammatory response and exerted a neuroprotective effect in the brains of tMCAO rats in vivo. The underlying mechanism is likely mediated through the inhibition of α7nAChR‐ERK signaling, which subsequently attenuates TLR4 and STAT3 pathways, thereby reducing microglial activation and M1‐type polarization while promoting M2‐type polarization (Figure 9). This study elucidates the target and potential mechanism of AD16 in the treatment of ischemic stroke, providing a scientific foundation for its clinical application.

## Author Contributions

Tao Chen, Xiao‐Lu Tang, and Zhi‐Hua Huang participated in the design of this study and performed the statistical analysis. Guo‐Jian Zhao carried out the study and drafted the manuscript. Li‐Mei Zhang, Mei Yang, Jia‐Hao Jiang, Si‐Rou Wang, Bo‐Xiang Yuan and Cheng Huang provided assistance for data acquisition, data analysis, and statistical analysis. All authors have read and approved the final manuscript.

## Ethics Statement

Animal experimental protocols were approved by the Gannan Medical University Animal Care and Use Committee. All animal experiments were performed following the Guidelines for the Care and Use of Laboratory Animals of Gannan Medical University (No. 2023576).

## Conflicts of Interest

The authors declare no conflicts of interest.

## Data Availability

The data that support the findings of this study are available from the corresponding author upon reasonable request.
